# Gouda cheese spoilage prevention: Biodegradable coating induced by *Bunium persicum* essential oil and lactoperoxidase system

**DOI:** 10.1002/fsn3.888

**Published:** 2019-02-10

**Authors:** Morteza Saravani, Ali Ehsani, Javad Aliakbarlu, Zahra Ghasempour

**Affiliations:** ^1^ Department of Food Hygiene and Quality Control Urmia University Urmia Iran; ^2^ Department of Food Science and Technology Tabriz University of Medical Science Tabriz Iran

**Keywords:** *Bunium persicum*, coating, Gouda cheese, lactoperoxidase

## Abstract

This study aimed to prepare an inhibitory edible coating for Gouda cheese based on whey protein containing lactoperoxidase system (LPOS) and *Bunium persicum* essential oil (EO) in order to control postpasteurization contamination. Using a full factorial design, the effects of LPOS and EO on microbiological characteristics and chemical indices of manufactured Gouda cheeses were evaluated during 90 days of storage time. *Listeria*,* lactic acid bacteria*,* Enterobacter*,* Escherichia*, and *Pseudomonas* species were considered as potential pathogenic and spoilage indicators of produced Gouda cheese samples. Chemical properties of cheeses were assessed using the free fatty acid, peroxide value, and thiobarbituric acid experiments. The results showed that bacteria counts remained constant in cheese samples coated with EO and also EO–LPOS. However, the survival of gram‐positive lactic acid bacteria and *Enterobacter* spp. was more pronounced in LPOS‐based coating. The most effective treatments on oxidation stability parameters in cheese samples were EO‐ and EO–LPOS coatings. By the addition of *B. persicum* EO and LPOS, further inhibition of the lipid oxidation of the cheese samples was achieved. Lipolysis, as a result of lipid degradation, was more pronounced in the control, whey‐coated, and whey–LPOS‐coated samples in comparison with whey–EO‐ and whey–EO–LPOS‐coated samples during the final days of storage time. These results indicate that antibacterial, lipid oxidation, and oxygen barrier properties of the coatings were developed by the addition of *B. persicum* EO and LPOS.

## 1 INTRODUCTION

Gouda is known as a yellow Dutch‐type cheese which is made from fresh cow's milk. It is one of the most popular cheese types in the world. Many biochemical (e.g., pH and water activity) and microbiological changes occur during the ripening period, contributing to the change in the quality of Gouda cheese. Microorganisms spoiling the dairy products differ widely because of the potential effects of initial preparation, formulation, processing, packaging, storage, and handling conditions. Furthermore, washed curd‐type cheeses are susceptible to proliferation of pathogens, especially coliforms; therefore, there is a high demand to monitor and control the quality of cheese during storage (Ledenbach & Marshall, 2009; Wemmenhove, Wells‐Bennik, Stara, Van Hooijdonk, & Zwietering, 2016).

Nowadays the utilization of coating in cheese packaging is one of the common approaches to preserve the quality of cheese during shelf life. Furthermore, nature‐ripened Gouda cheeses are coated regularly during ripening and storage time (Wemmenhove et al., 2016). Different natural components including polysaccharides, proteins, and lipids (alone or in combination) can be utilized as edible coating materials. The efficiency of an edible coating in preserving food quality is mainly related to its moisture and gas barrier properties (Aloui & Khwaldia, 2016). Whey proteins, a biodegradable coatings and films material, are a major by‐product of the cheese industry. Heat‐denatured whey proteins produce transparent and flexible films with excellent water vapor, gas and oil barrier properties (Ramos, Fernandes, Silva, Pintado, & Malcata, 2012). Incorporating antimicrobial compounds into biopolymer edible coatings could improve the quality and shelf life of food products (Aloui & Khwaldia, 2016). Rather than incorporating the antimicrobial agents directly into food, blending them in film or coating solutions induces a functional effect on the food surface. Due to the consumers’ concerns about health, there is particular interest in food industry to use natural food preservatives such as antimicrobial enzymes and bacteriocins (Shokri, Ehsani, & Jasour, 2015).

*Bunium persicum (B. persicum)* or black cumin is a plant from the Apiaceae family which might have been originated in the area between central Asia and northern India. *B. persicum* seed, which is called “zireh kuhi,” “zireh kermani,” or “zireh siyah,” is utilized as a culinary spice. Generally, the main components of *B. persicum* are cuminaldehyde; p‐mentha‐1,3‐dien‐7‐al; p‐mentha‐1,4‐dien‐7‐al (=c‐terpinene‐7‐al); and terpene hydrocarbons including c‐terpinene, p‐cymene, β‐pinene, and limonene (Mortazavi, Eikani, Mirzaei, Jafari, & Golmohammad, 2010). Few researches have employed essential oils (EOs) as natural food preservatives against fungal and bacterial pathogens from different types of cheese: traditional cheese (Philippe, Souaïbou, Paulin, Issaka, & Dominique, 2012; Philippe, Souaïbou, Guy, et al., 2012), white‐brined cheese (Ehsani & Mahmoudi, 2012; Mehdizadeh, Narimani, Mojaddar Langroodi, Moghaddas Kia, & Neyriz‐Naghadehi, 2018; Sadeghi, Mohammadi, Jamilpanah, Bashiri, & Bohlouli, 2016), coalho cheese (Ribeiro, Siqueira, da Silva Velozo, & Guimarães, 2013), and soft cheese (Smith‐Palmer, Stewart, & Fyfe, 2001). Antifungal activity of *B. persicum* against different molds and yeasts species may present a further promising usage of this plant. Consequently, the application of this medicinal plant EO could be an alternative of chemical antimicrobial preservatives in edible packagings.

Furthermore, lactoperoxidase system (LPOS), an antimicrobial enzyme having a broad antimicrobial spectrum, is an effective agent in biological systems such as milk, saliva, and tears of mammals. This enzyme often has bactericidal effects on gram‐negative bacteria and bacteriostatic effects on gram‐positive bacteria. Also, it has antifungal and antiviral activities. Three components of LPOS consist of lactoperoxidase (LPO) enzyme, thiocyanate, and hydrogen peroxide (H_2_O_2_). Lactoperoxidase oxidation of thiocyanate (SCN−) occurs by using hydrogen peroxide and produces intermediate antimicrobial materials such as hypothiocyanite (OSCN−) and hypothiocyanous acid (HOSCN). These materials have the potential of inhibiting the microorganisms’ growth by oxidizing sulfhydryl (–SH) groups in their enzyme systems (Munsch‐Alatossava, Gursoy, Lorilla, Gauchi, & Alatossava, 2018; Yener, Korel, & Yemenicioğlu, 2009). Min, Harris, and Krochta (2005) reported complete inhibition of *Salmonella enterica* and *Escherichia coli* O157:H7 (4 log CFU/cm) using LPOS in whey protein‐based film. Shokri et al. (2015) also applied LPOS–whey protein coating for extension of rainbow trout fillets’ shelf life.

The main objective of this study was to investigate the effects of edible coatings containing LPOS and *B. persicum* EO as antimicrobial agents on the quality indices and microbial characteristics of Gouda cheese during storage.

## 2 MATERIALS AND METHODS

### 2.1 Materials

Gouda cheese was obtained from Kaleh Co. (Iran). LPOS consisted of lactoperoxidase (LPO, 120 U/mg; Sigma‐Aldrich), glucose oxidase (Sigma‐Aldrich), potassium thiocyanate (Bioserae, France), hydrogen peroxide (Merck, Germany), and D‐glucose (Sigma‐Aldrich). The air‐dried seeds of *B. persicum* were supplied from Kerman Province (Iran) and confirmed by the Herbarium of West Azerbaijan Agricultural and Natural Resource Center, Urmia, Iran. Whey protein isolate (80% protein) was acquired from Serva Co. (Germany). Glycerol, as coating plasticizer, was obtained from Merck (Germany). *Listeria monocytogenes* (ATCC 19118) and lyophilized cultures of *E. coli* O157:H7 (ATCC 43894) were prepared from the culture collection of the Department of Food Hygiene and Quality Control, Urmia University, Urmia, Iran. Media for bacterial cultures including Plate Count Agar, de Man–Rogosa–Sharpe agar (MRS agar), Eosin Methylene Blue agar (EMB agar), PALCAM agar, Violet Red Bile Glucose agar, and Cetrimide Fucidin Cephaloridine agar were all obtained from Micromedia (Australia), and King Agar was purchased from Merck (Germany). All utilized reagents were of analytical grade.

### 2.2 Gouda cheese preparation

In this research, the required treatments were based on five coating formulations which were assigned randomly during the study:

1. Control: 0% EO–LPOS (C)
2. Whey protein coating (W)
3. Whey protein coating containing 5% LPOS (WL)
4. Whey protein coating containing 0.5% EO (WE)
5. Whey protein coating containing 5% LPOS and 0.5% EO (WLE).

Cheese slices were dipped in the well‐stirred coating solution for 60 s. The ratio of cheese to the solution was 1:2. After taking away the immersed cheese samples from the solution, they were drained well, packed in polyethylene bags, and kept at 4±1°C for 90 days.

#### 2.2.1 Extraction of *B. persicum* EO

Initially, dried seeds (100 g) were ground into powder in a grinder, and then by using a Clevenger‐type apparatus, they were exposed to steam distillation for 2.5 hr. In the next step, the obtained EO was well drained from water and dried over anhydrous sodium sulfate until the last traces of moisture were evaporated. At last, the substance was stored in dark glass bottles at 4°C for more experiments (Ehsani, Hashemi, Naghibi, Mohammadi, & Khalili Sadaghiani, 2016). The characteristics of essential oil were determined by GC/MS (30 m × 250 μm × 0.25 μm).

#### 2.2.2 Bacterial inoculation

Lyophilized cultures of *E. coli* (ATCC 43894) and *L. monocytogenes* (ATCC 19118) were obtained from the culture collection of the Department of Microbiology, Faculty of Veterinary Medicine, Urmia, Iran, and inoculated on the surface of cheese samples before coating with the dilution of 10^6^ and 10^3^ (CFU/ml), respectively.

#### 2.2.3 Preparation of LPOS

The weight ratios of LPOS components for lactoperoxidase, glucose oxidase, glucose, potassium thiocyanate, and hydrogen peroxide were 1.00, 0.35, 108.70, 1.09, and 2.17, respectively. Following that, the components were dissolved separately in 50 ml phosphate buffer (pH 6.2), and then, the concentrations of the components were altered on the basis of 15.5 mg LPO (Cissé, Montet, Tapia, Loiseau, & Ducamp‐Collin, 2012). To increase the antimicrobial activity of LPOS, the solution was incubated at 23°C for 24 hr under vibration at 160 rpm (Shokri et al, (2015).

#### 2.2.4 Preparation of whey protein solution

In order to prepare whey protein solution (WPS), 10 g of whey protein was mixed with 100 ml of distilled water and stirred at a controlled temperature of 90°C until a clear mixture was obtained. Just the same amount of whey protein, glycerol was added to the solution. The amount of LPOS in the whey protein solution was 5% (v/v) (Shokri et al, (2015). In the whey protein solution, the 0.5% v/v concentration of *B. persicum* EO was applied as coating‐forming solution.

### 2.3 Bacteriological analysis

Initially, 25 g of cheese samples was brought to a final volume of 250 ml with 0.1% peptone water and then homogenized in a stomacher (Pulsifier^®^, UK) for 1 min. Dilution process was done serially in all samples. The method of drop plate by using a proper tool was applied to count the number of all bacteria.

Total viable counts were fulfilled in Plate Count Agar for 48 hr at 30°C. *Pseudomonas* spp. were incubated for 24–48 hr at 23°C on Cetrimide Fucidin Cephaloridine agar. *Enterobacteriaceae* count was done with Violet Red Bile Glucose agar using the overlay method after incubation for 24 hr at 37°C. Lactic acid bacteria (LAB) were enumerated by de Man–Rogosa–Sharpe agar (MRS agar) supplemented with 0.1 g/L cycloheximide after incubation for 5 days at 25°C under anaerobic conditions (Alizadeh Sani, Ehsani, & Hashemi, 2017). Psychotropic bacteria were determined using King Agar after incubation for 48 hr at 21°C. For the bacterial count, dilutions were prepared for each bacterium and plated on PALCAM agar and EMB agar for *L. monocytogenes* and *E. coli* O157:H7, respectively.

### 2.4 Chemical analysis

#### 2.4.1 pH

Through using a pH meter (Eutech^®^ CyberScan, pH 510, Singapore), the pH value for the homogeneous mixture of distilled water (1:10, w/v) and cheese samples was measured.

#### 2.4.2 Lipid extraction

During storage days (0, 7, 15, 30, 60, and 90), 10 g of each cheese sample was mixed for 2 min in 20 ml chloroform and 40 ml methanol. Then, 20 ml chloroform and 20 ml methanol were added to the homogenized mixture consecutively. Vacuum filtration using Whatman No. 1 filter paper was applied to separate solid from liquid. After preparing a transparent solution, the solvent was removed by rotary evaporator (Bligh & Dyer, 1959). The prepared lipid samples were sealed in opaque bottles and stored at −80°C until use.

#### 2.4.3 Thiobarbituric acid

First, 0.2 g of cheese fat was homogenized with 25 ml of butanol solution. The next step was to blend 5 ml of the prepared solution and 5 ml of TBA reagent (200 mg TBA in 100 ml butanol) and heat them in a boiling water bath for 2 hr. Then, they were cooled under running water for 1 min and the absorbance was measured at 539 nm against a blank (consisting of 5 ml of TBA reagent and 5 ml butanol). The values of TBA were stated as milligram malonaldehyde (MA) per kilogram of sample (Shin, Song, Seo, & Song, 2012).

#### 2.4.4 Free fatty acids

A 0.2 g of fat extracted from the Gouda cheese was dissolved in 50 ml solvent (1:1 ethanol and diethyl ether). The total mixture was titrated with 0.05 N alcoholic KOH solution. The results were expressed as milligram KOH per gram of cheese fat.

(1) $$\text{Acid}\;\text{value}=\frac{56.1\times N\times V}{w}$$

N= KOH normality; V= volume of KOH; and w= lipid mass

(2) $$\text{FFA}=\text{acid}\;\text{value}\times \frac{1}{2}$$

(Hayaloglu, 2007).

#### 2.4.5 Peroxide value

First, 0.1 g cheese fat was weighed into a 250‐ml flask and 25 ml acetic acid/chloroform (ratio of 3:2, v/v) was poured. Then, the mixture was stirred up to complete dissolution of remained lipids. After potassium iodide (1 ml) addition, the solution was maintained in a dark room for 10 min. Distilled water (20 ml) was poured and titrated with sodium thiosulfate, with 1.5% starch as an indicator. The peroxide value was represented as meq peroxide/kg cheese sample (Shin et al., 2012).

### 2.5 Sensory analysis

To perform the sensory evaluations, 10 trained panelists who were familiar with odor, color, and overall acceptability of cheese were selected. An acceptance test using a 9‐point hedonic scale was used to evaluate the overall acceptance, with 1 as “dislike extremely” and 9 as “like extremely.” Water was used for mouth rinsing between evaluations of samples (Cui, Wu, Li, & Lin, 2017).

### 2.6 Statistical analysis

A completely randomized factorial design, three replicates, was proposed with two independent factors: cheese type and storage time (Table 1). Data were subjected to one‐way analysis of variance (ANOVA) using Design‐Expert 10 software to determine the significant difference among samples. The differences were considered significant when *p* < 0.05.

**Table 1:** Sensory evaluation scores of different cheese treatments during 90 days of storage time

Run	Storage time (day)	Cheese type	Odor	Color	Overall acceptance (from 9)
1	0	C	8.74	8.83	8.8
2	7	C	7.63	8.61	8.63
3	15	C	6.46	7.42	7.19
4	30	C	5.42	5.34	5.41
5	60	C	3.54	3.14	3.44
6	90	C	2.70	2.25	2.63
7	0	W	8.76	8.82	8.82
8	7	W	7.57	8.55	8.57
9	15	W	6.53	7.49	7.19
10	30	W	5.44	5.36	5.43
11	60	W	3.61	3.21	3.51
12	90	W	2.61	1.94	2.47
13	0	WL	8.71	8.83	8.77
14	7	WL	7.66	8.64	8.66
15	15	WL	6.51	7.50	7.21
16	30	WL	5.48	5.46	5.53
17	60	WL	3.85	3.45	3.52
18	90	WL	2.65	2.48	2.51
19	0	WE	8.74	8.84	8.81
20	7	WE	7.71	8.71	8.73
21	15	WE	6.46	7.5	7.24
22	30	WE	5.63	5.39	5.46
23	60	WE	4.2	4.19	3.49
24	90	WE	2.83	2.4	2.53
25	0	WLE	8.71	8.81	8.78
26	7	WLE	7.69	8.87	8.89
27	15	WLE	6.51	7.55	7.19
28	30	WLE	5.54	5.46	5.53
29	60	WLE	4.23	4.25	3.62
30	90	WLE	3.25	2.52	2.68

*Note*

C: 0% lactoperoxidase (LPOS) and essential oil (EO); W: whey protein coating; WL: whey protein coating containing 5% LPOS; WE: whey protein coating containing 0.5% EO; WLE: whey protein coating containing 5% LPOS and 0.5% EO.

## 3 RESULTS AND DISCUSSION

### 3.1 Bacterial culture and total count

Aerobic psychotropic gram‐negative bacteria, enteropathogenic bacteria, heterofermentative lactobacilli, and spore‐forming bacteria are known as spoilage microorganisms in dairy products. Cheese formulation, processing, packaging, storage, distribution, besides intrinsic conditions like moisture content , pH and storage time temperature regulate the spoilage rate in cheese products (Khorshidian, Yousefi, Khanniri, & Mortazavian, 2017; Ledenbach & Marshall, 2009).

**Figure 1:**
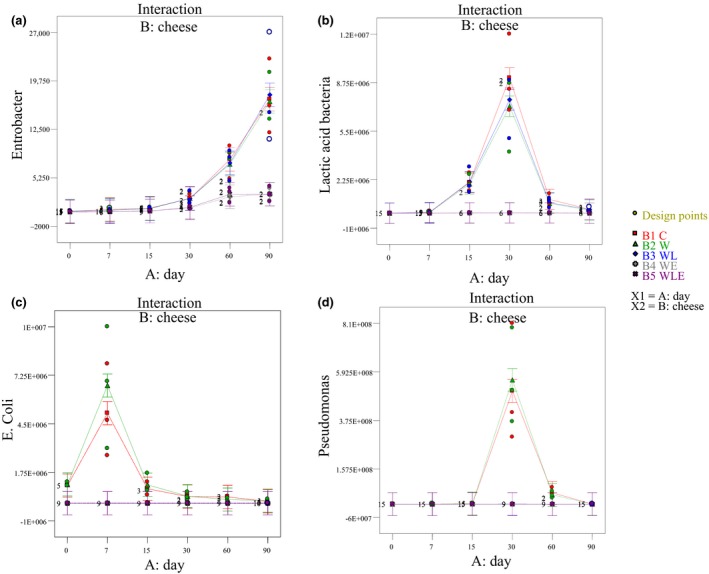
Effect of cheese type × storage time interaction on bacterial growth (C: 0% lactoperoxidase (LPOS) and essential oil (EO); W: whey protein coating; WL: whey protein coating containing 5% LPOS; WE: whey protein coating containing 0.5% EO; WLE: whey protein coating containing 5% LPOS and 0.5% EO)

In this study, storage time and type of cheese had significant effects on microbiological activity in the control cheese and coated samples (*p* < 0.05) (Figure 1). According to the results, the activity of some considered species started during the initial days of storage (*E. coli*,* Listeria*, and *LAB*) and that of the others increased at day 30 of storage in the control cheese and the whey protein‐coated samples. As *L. monocytogenes* can survive over a wide pH range and high salt amounts (i.e., pH: 5.00–6.50; *a*_w_: 0.94–0.99; moisture: soft, semihard, hard), it is remarked as the main risk for cheese industries. Gas‐forming microorganisms in Gouda‐type cheese are heterofermentative lactobacilli and *Clostridium tyrobutyricum*. These microorganisms can rapidly degrade lactose and subsequently lead to formation of lactate, acetate, ethanol, and CO_2_ in approximately identical ratios (Ledenbach & Marshall, 2009). Postpasteurization contamination is mainly due to the presence of coliform in cheese which is brought by unsanitary conditions during cheese processing (Kwenda, Nyahada, Musengi, Mudyiwa, & Muredzi, 2014; Trmčić et al., 2016).

Cheese samples expel moisture, particularly at the beginning of ripening, leading to bacterial growth. It is also reported that NaCl reduction, due to diffusion into cheese matrix, results in spore‐forming bacteria outgrowth. However, the overall moisture of Gouda cheese decreases steadily during ripening, which avoids bacterial growth. Besides, some microorganisms such as coliforms grow as long as lactose, or citrate, is present (Fox, McSweeney, Cogan, & Guinee, 2004; Jo, Benoist, Ameerally, & Drake, 2018).

Considering the data analyses, except for LAB and Enterobacter (Figure 1a and b), the LPOS‐coated cheese samples showed antibacterial activity up to the end of the 90‐day storage period. The number of all bacteria was steady in all types of cheeses during storage in both the EO‐ and EO–LPOS‐coated cheese samples. The enzyme LP shows the most bactericidal efficacy against gram‐negative bacteria, while the most bacteriostatic efficacy is related to gram‐positive bacteria (Yener et al., 2009). According to Munsch‐Alatossava et al.'s (2018) findings, a gram‐positive bacterium will be less susceptible to the antibacterial effects of OSCN– (hypothiocyanite in its base form), because of the presence of cell wall outside the cytoplasmic membrane, so lower amounts of HOSCN (hypothiocyanite in its acid form) will leach into the cytoplasm and finally result in bacteriostatic effects. The outer membrane pores of gram‐negative bacteria will allow the negatively charged OSCN– to penetrate the periplasmic space together with the neutral HOSCN, which is able to leach through outer membrane and cytoplasmic membrane. Finally, high intracellular concentrations of HOSCN/OSCN– will be reached and lead to more bactericidal effects in cytoplasmic area and protein dysfunctions eventually. Several bacterial species have the ability to eliminate or neutralize free HOSCN/OSCN– molecules and make themselves more resistant to LPOS (Munsch‐Alatossava et al., 2018; Renye & Somkuti, 2015). The degree of resistance to LPOS is species‐dependent; however, incubation conditions such as aerobiosis/anaerobiosis and the growth phase stage render bacteria more or less susceptible to LPOS; gram‐negative catalase‐positive bacteria, such as *Pseudomonas* spp. and coliforms, may be killed if H_2_O_2_ is supplied exogenously; and gram‐positive catalase‐negative bacteria, such as streptococci or lactobacilli, are generally inhibited, but not killed, by the LPOS (Munsch‐Alatossava et al., 2018). According to these, LPOS is generally used in combination with other preservation methods.

Despite controversies about the antimicrobial mechanisms of EOs, several targets in the microorganisms are identified as the sites of the EOs’ action (Nazzaro, Fratianni, De Martino, Coppola, & De Feo, 2013). Although it has been reported that EOs are more effective against gram‐positive bacteria in comparison with gram‐negative bacteria (Hyldgaard, Mygind, & Meyer, 2012; Seow, Yeo, Chung, & Yuk, 2014), our results have shown similar trends in all species; thus, the growth of all bacteria was inhibited. As gram‐negative microorganisms have an outer membrane rich in lipopolysaccharide, they could prevent entry of various antimicrobial components of essential oil. The presence of hydrolytic enzymes in the periplasmic space causes degradation of antimicrobial substances. On the other hand, according to the absence of periplasmic space in gram‐positive bacteria, the hydrophobic essential oils can pass directly through phospholipid bilayer of the cell membrane and induce ion increment permeability, intracellular constituent leakages, and impedance of enzyme systems (Seow et al., 2014).

Ehsani et al. (2016) evaluated the antibacterial properties of *B. persicum* against *Salmonella typhimurium*,* E. coli* O157:H7, *Staphylococcus aureus*, and *L. monocytogenes* and the antibacterial effects of the oil on survival of *L. monocytogenes* and *E. coli* O157:H7 in Iranian white cheese. They indicated that high susceptibility of gram‐positive bacteria and relative sensitivity of gram‐negative bacteria are due to pronounced decrement of *E. coli* O157:H7 and *L. monocytogenes* in manufactured white cheeses during 45 days of storage time (Ehsani et al., 2016). Furthermore, there are several previous reports about the antimicrobial effects of other EOs, including those of *Ocimum gratissimum* against *Aspergillus flavus*,* Aspergillus tamarii*,* Fusarium poae*,* Fusarium verticillioides*,* Penicillium citrinum*, and *Penicillium griseofulvum* (Philippe, Souaïbou, Guy, et al., 2012); *Cinnamomum zeylanicum* and *O. gratissimum* against *Aspergillus terreus*,* Aspergillus ustus*,* Aspergillus niger*,* Aspergillus aculeatus*,* Penicillium brevicompactum*, and *Scopulariopsis brevicaulis* (Philippe, Souaïbou, et al., 2012); *Cuminum cyminum* L. plus *Lactobacillus acidophilus* against *S. aureus* (Sadeghi, Akhondzadeh Basti, Noori, Khanjari, & Partovi, 2013); rosemary and *Pimpinella anisum* against *E. coli* (Ehsani & Mahmoudi, 2012; Ribeiro et al., 2013); and *Mentha spicata*,* Mentha pulegium*,* Pimenta racemosa*,* Syzygium aromaticum*,* Cinnamomum verum*, and *Thymus vulgaris* against *Listeria* spp. (Smith‐ Smith‐Palmer et al., 2001; Moosavi, Esmaeili, & Mostafavi, 2013; Sadeghi et al., 2016). All of the above‐mentioned reports demonstrated that the EOs extracted from plants have good antimicrobial properties and can be used in cheese production.

### 3.2 TBARS, POV, pH, and FFA

The peroxide value was measured to assess the lipid oxidation of Gouda cheese during storage. The antiperoxidation evaluation was followed by an adaptation of the TBARS assay, which relies on the colorimetric detection of the malondialdehyde formation by polyunsaturated lipid degradation as a result of reactive oxygen species. Eventually reaction of the malondialdehyde with thiobarbituric acid (TBA) represents a colored compound.

**Figure 2:**
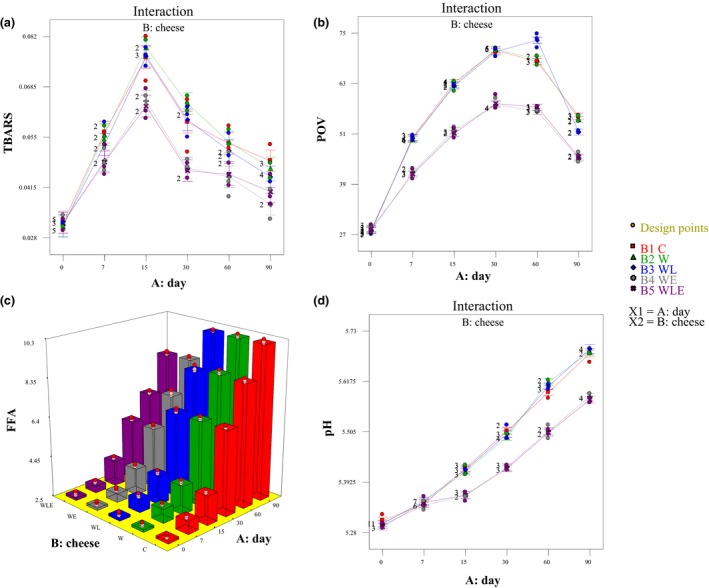
Effects of cheese type and storage time on the thiobarbituric acid (TBARS), peroxide value (POV), pH, and free fatty acid (FFA) content

The ANOVA results revealed that cheese type × storage time interaction had a significant effect on TBARS and POV (*p* < 0.05). The trend shows that both oxidation indicators increased until day 30. Also, the following decrement of TBARS and POV during storage had no association with cheese types, except in POV of WL cheese which showed a significant correlation between storage time and POV even at 60 days (Figure 2a and b). The reduction in POVs was also reported in an investigation by Lee, Yang, and Song (2016), who prepared packaging of Gouda cheese based on fish skin gelatin containing *Moringa oleifera Lam*. leaf extract. The decrement of TBARS and POV is a consequence of unstable peroxide decomposition to secondary products such as ketones, hydrocarbons, and aldehydes. Our results showed that the fabricated coating on the Gouda cheese could effectively inhibit the production of primary lipid oxidation products in this cheese type. Our results are similar to those of Nascimento et al. (2015), where the *M. oleifera Lam*.‐containing fish oil had lower POV than those with other synthetic antioxidants during storage.

A similar trend in the TBARS assessment was obtained in all treatments. Lee et al. (2016) showed that the permeability to gases such as oxygen is one of the crucial parameters of films that may govern the lipid oxidation of the cheese. So probably cheese samples that were coated with the *B. persicum* EO accompanied by LPOS film had less contact with oxygen. These results reveal that the oxygen barrier property of the coating was intensified by the addition of *B. persicum* EO and LPOS, in which further inhibition of the lipid oxidation of the cheese by decreasing the oxygen content in the packaging was achieved.

The effects of cheese type × storage time interaction on the pH and FFA content were similar and are presented in Figure 2c and d. As demonstrated, during storage, the pH and FFA content were also significantly increased (*p* < 0.001), which is most apparent in the WL‐type cheese. The treatments did not change the pH value of Gouda cheese during storage (Figure 2d). The pH ranged between 5.28 and 5.61 during 90 days of storage time, which represents the poor postacidification process of samples. pH reduction during the ripening period is mainly due to the metabolism of the retained lactose to lactic acid by nonstarter lactic acid bacteria. Higher levels of dissolved CO_2_ in the cheese atmosphere enable buffering activity according to the lower CO_2_ permeability. Substantially, increment of pH of the cheese samples is the consequence of alkaline compound formation due to protein degradation by proteolytic enzymes during the ripening period in the WL and WE samples. Fatty acids constitute over 90% of total edible fats and oils. There are many naturally occurring fatty acids with very different chemical and physical characteristics. Free fatty acids (FFA) are likely to contribute to the flavor and odor of Gouda cheese (Sulieman, Ohag, Hassan, Alreshidi, & Veettil, 2018). Figure 2c demonstrates the mean value of fatty acid composition of laboratory‐made Gouda expressed as g/100 g of the total fatty acids. The results showed that FFA production rate is increasing during storage, which is due to the lipolysis phenomenon. Milk fat hydrolysis which is called as lipolysis is a complex phenomenon which is the results of the activity of natural lipases of the milk, the lipolytic enzymes of the starter and non‐starter lactic acid bacteria, and lipases of the psychrotrophs during cheese manufacture and storage time (Soleimani‐Rambod, Zomorodi, Naghizadeh Raeisi, Khosrowshahi Asl, & Shahidi, 2018). The amounts of free fatty acids (FFA) were used as indicators of lipolysis in the cheese samples. The free fatty acid composition of samples was significantly affected by edible coatings. Lipolysis was more pronounced in the C‐, W‐, and WL‐type cheeses during the final days of storage time. The lower amount of WE and WLE FFA content could be related to the inhibitory effect of different applied coatings on the lipolysis rate of Gouda cheese samples.

### 3.3 Sensory evaluation

Sensory characteristics such as odor, texture, and appearance play an important role in the overall acceptability from the consumers’ point of view. Thus, consumers are not engaged in consideration of a functional food if the incorporated ingredients confer unpleasant flavor (Moghaddas Kia, Alizadeh, & Esmaiili, 2018; Moghaddas Kia, Ghasempour, Ghanbari, Pirmohammadi, & Ehsani, 2018).

Proteolysis and lipolysis play an important role in the sensory of special Gouda‐type cheese. To a large extent, the ripening procedures are catalyzed by (residual) milk and rennet enzymes, and by enzymes from (adjunct) starters and possible nonstarter lactic acid bacteria present in the cheese. Under these conditions, casein is degraded by residual chymosin, and to a lesser extent by plasmin and by proteinases, into larger peptides. These are further degraded, finally yielding free amino acids and amino acid degradation products. Peptides, amino acids (Moghaddas Kia, et al., 2018; Moghaddas Kia, Ghasempour, et al., 2018), and (volatile) components, also from fat (Sulieman et al., 2018) and citrate conversion, together determine the full flavor (taste and aroma) of Gouda cheese (Fox et al., 2004). Sensory evaluation scores are presented in Table 1. Our findings about the positive sensory effects of using *B. persicum* EO in cheese preparation are in line with those reported previously by Ehsani et al. (2016) and Hassanien, Mahgoub, and El‐Zahar (2014). They found that *B. persicum* EO‐enriched cheese samples had significantly higher color, odor, flavor, texture, and general acceptability scores than control cheese (Ehsani et al., 2016). Also, Hassanien et al. (2014) found that Domiati soft cheeses supplemented with black cumin oil showed higher sensory scores compared to control cheese (Hassanien et al., 2014). On the other hand, it has been reported that lactoperoxidase system (LPOS), alone and in combination with some plants and their derivatives, can provide beneficial effects for the food industry. For instance, Sharifi, Khanzadi, Hashemi, and Azizzadeh (2017) reported that *Zataria multiflora* Boiss EO and LPOS, when used in combination in alginate solution, had stronger antibacterial effects against *L. monocytogenes* and *E. coli* O157:H7 in rainbow trout fillets during 16 days of storage in comparison with each alone.

## 4 CONCLUSIONS

Based on our findings in the present study, *B. persicum* EO was very effective against *LAB*,* Listeria*, and *Enterobacter* growth unlike LPOS‐containing coatings. LPOS is recommended to be used in combination with other preservation methods, as it was effective in inhibiting gram‐negative bacteria. Decreases in lipid oxidation indices and also free fatty acid content were more pronounced in whey protein coatings involving EO and also EO–LPOS. LPOS as an active gram‐negative microorganism inhibitor and EO as a gram‐positive bacterium inhibitor as well as an oxidation inhibitor are seriously recommended as preservative agents against chemical and microbiological spoilage in cheese. Therefore, *B. persicum* EO in combination with LPOS can be applied as a natural antimicrobial agent for extending the shelf life of washed and ripened cheeses.

## CONFLICT OF INTEREST

The authors declare that they do not have any conflict of interest.

## ETHICAL REVIEW

This study does not involve any human or animal testing.

## INFORMED CONSENT

Written informed consent was obtained from all study participants. The manuscript has not been published previously (partly or in full) or submitted for publication elsewhere.

## References

[fsn3888-bib-0001] Alizadeh Sani, M. , Ehsani, A. , & Hashemi, M. (2017). Whey protein isolate/cellulose nanofibre/TiO_2_ nanoparticle/rosemary essential oil nanocomposite film: Its effect on microbial and sensory quality of lamb meat and growth of common foodborne pathogenic bacteria during refrigeration. International Journal of Food Microbiology, 251, 8–14. 10.1016/j.ijfoodmicro.2017.03.018 28376399

[fsn3888-bib-0002] Aloui, H. , & Khwaldia, K. (2016). Natural antimicrobial edible coatings for microbial safety and food quality enhancement. Comprehensive Reviews in Food Science and Food Safety, 15(6), 1080–1103. 10.1111/1541-4337.12226 33401837

[fsn3888-bib-0003] Bligh, E. G. , & Dyer, W. J. (1959). A rapid method of total lipid extraction and purification. Canadian Journal of Biochemistry and Physiology, 37(8), 911–917. 10.1139/y59-099 13671378

[fsn3888-bib-0004] Cissé, M. , Montet, D. , Tapia, M. S. , Loiseau, G. , & Ducamp‐Collin, M. N. (2012). Influence of temperature and relative humidity on the immobilized lactoperoxidase system in a functional chitosan film. Food Hydrocolloids, 28(2), 361–366. 10.1016/j.foodhyd.2012.01.012

[fsn3888-bib-0005] Cui, H. , Wu, J. , Li, C. , & Lin, L. (2017). Improving anti‐listeria activity of cheese packaging via nanofiber containing nisin‐loaded nanoparticles. LWT – Food Science and Technology, 81, 233–242. 10.1016/j.lwt.2017.04.003

[fsn3888-bib-0006] Ehsani, A. , Hashemi, M. , Naghibi, S. S. , Mohammadi, S. , & Khalili Sadaghiani, S. (2016). Properties of *Bunium persicum* essential oil and its application in Iranian white cheese against *Listeria monocytogenes* and *Escherichia coli* O157:H7. Journal of food safety, 36(4), 563–570. 10.1111/jfs.12277

[fsn3888-bib-0007] Ehsani, A. , & Mahmoudi, R. (2012). Phytochemical properties and hygienic effects of *Allium ascalonicum* and *Pimpinella anisum* essential oils in Iranian white brined cheese. Journal of Essential Oil Bearing Plants, 15(6), 1013–1020. 10.1080/0972060X.2012.10662606

[fsn3888-bib-0008] Fox, P. F. , McSweeney, P. L. , Cogan, T. M. , & Guinee, T. P. (Eds.) (2004). Cheese: Chemistry, physics and microbiology: General aspects, chapter 34: Gouda and Related Cheeses. Elsevier.

[fsn3888-bib-0009] Hassanien, M. F. R. , Mahgoub, S. A. , & El‐Zahar, K. M. (2014). Soft cheese supplemented with black cumin oil: Impact on food borne pathogens and quality during storage. Saudi Journal of Biological Sciences, 21(3), 280–288. 10.1016/j.sjbs.2013.10.005 24955014 PMC4061416

[fsn3888-bib-0010] Hayaloglu, A. A. (2007). Comparisons of different single‐strain starter cultures for their effects on ripening and grading of Beyaz cheese. International Journal of Food Science & Technology, 42(8), 930–938. 10.1111/j.1365-2621.2006.01312.x

[fsn3888-bib-0011] Hyldgaard, M. , Mygind, T. , & Meyer, R. L. (2012). Essential oils in food preservation: Mode of action, synergies, and interactions with food matrix components. Frontiers in Microbiology, 3(12), 1–24. 22291693 10.3389/fmicb.2012.00012 PMC3265747

[fsn3888-bib-0012] Jo, Y. , Benoist, D. M. , Ameerally, A. , & Drake, M. A. (2018). Sensory and chemical properties of Gouda cheese. Journal of Dairy Science, 101(3), 1967–1989. 10.3168/jds.2017-13637 29274971

[fsn3888-bib-0013] Khorshidian, N. , Yousefi, M. , Khanniri, E. , & Mortazavian, A. M. (2017). Potential application of essential oils as antimicrobial preservatives in cheese. Innovative Food Science & Emerging Technologies, 45, 62–72.

[fsn3888-bib-0014] Kwenda, A. , Nyahada, M. , Musengi, A. , Mudyiwa, M. , & Muredzi, P. (2014). An investigation on the causes of *Escherichia coli* and coliform contamination of cheddar cheese and how to reduce the problem (A case study at a cheese manufacturing firm in Harare, Zimbabwe). International Journal of Nutrition and Food Sciences, 3(3), 6–14. 10.11648/j.ijnfs.s.2014030601.12

[fsn3888-bib-0015] Ledenbach, L. H. , & Marshall, R. T. (2009). Microbiological spoilage of dairy products In Compendium of the microbiological spoilage of foods and beverages (pp. 41–67). New York, NY: Springer 10.1007/978-1-4419-0826-1

[fsn3888-bib-0016] Lee, K. Y. , Yang, H. J. , & Song, K. B. (2016). Application of a puffer fish skin gelatin film containing *Moringa oleifera* Lam. leaf extract to the packaging of Gouda cheese. Journal of Food Science and Technology, 53(11), 3876–3883. 10.1007/s13197-016-2367-9 28035143 PMC5156630

[fsn3888-bib-0017] Mehdizadeh, T. , Narimani, R. , Mojaddar Langroodi, A. , Moghaddas Kia, E. , & Neyriz‐Naghadehi, M. (2018). Antimicrobial effects of *Zataria multiflora* essential oil and *Lactobacillus acidophilus* on *Escherichia coli* O157 stability in the Iranian probiotic white‐brined cheese. Journal of Food Safety, 38, e12476 10.1111/jfs.12476

[fsn3888-bib-0018] Min, S. , Harris, L. J. , & Krochta, J. M. (2005). Antimicrobial effects of lactoferrin, lysozyme, and the lactoperoxidase system and edible whey protein films incorporating the lactoperoxidase system against *Salmonella enterica* and *Escherichia coli* O157:H7. Journal of Food Science, 70(7), m332–m338. 10.1111/j.1365-2621.2005.tb11476.x 16629020

[fsn3888-bib-0019] Moghaddas Kia, E. M. , Alizadeh, M. , & Esmaiili, M. (2018). Development and characterization of probiotic UF Feta cheese containing *Lactobacillus paracasei* microencapsulated by enzyme based gelation method. Journal of Food Science and Technology, 55(9), 3657–3664. 10.1007/s13197-018-3294-8 30150825 PMC6098783

[fsn3888-bib-0020] Moghaddas Kia, E. , Ghasempour, Z. , Ghanbari, S. , Pirmohammadi, R. , & Ehsani, A. (2018). Development of probiotic yogurt by incorporation of milk protein concentrate (MPC) and‎ microencapsulated *Lactobacillus paracasei*‎ in gellan‐caseinate mixture. British Food Journal, 120(7), 1516–1528. 10.1108/BFJ-12-2017-0668

[fsn3888-bib-0021] Moosavi, M. H. , Esmaeili, S. , & Mostafavi, E. (2013). Antibacterial effect of *Mentha spicata* essential oil on *Listeria monocytogenes* in traditional Lighvan cheese. Journal of Food Safety, 33(4), 509–514. 10.1111/jfs.12083

[fsn3888-bib-0022] Mortazavi, S. V. , Eikani, M. H. , Mirzaei, H. , Jafari, M. , & Golmohammad, F. (2010). Extraction of essential oils from *Bunium persicum* Boiss. using superheated water. Food and Bioproducts Processing, 88(2), 222–226. 10.1016/j.fbp.2009.11.005

[fsn3888-bib-0023] Munsch‐Alatossava, P. , Gursoy, O. , Lorilla, P. M. , Gauchi, J. P. , & Alatossava, T. (2018). Antibacterial effects and modes of action of the activated lactoperoxidase system (LPOS), of CO_2_ and N_2_ gas as food‐grade approaches to control bovine raw milk‐associated bacteria. Food Control and Biosecurity Handbook of Food Bioengineering, 519–541. 10.1016/B978-0-12-811445-2.00015-5

[fsn3888-bib-0024] Nascimento, J. A. , Magnani, M. , Sousa, J. , Arau′jo, K. L. , Epaminondas, P. S. , Souza, A. S. , … Souza, A. G. (2015). Assessment of the antioxidant effects of *Moringa oleifera* Lam. extract in fish oil during storage. Journal of Food Processing and Preservation, 40, 29–36.

[fsn3888-bib-0025] Nazzaro, F. , Fratianni, F. , De Martino, L. , Coppola, R. , & De Feo, V. (2013). Effect of essential oils on pathogenic bacteria. Pharmaceuticals, 6(12), 1451–1474. 10.3390/ph6121451 24287491 PMC3873673

[fsn3888-bib-0026] Philippe, S. , Souaïbou, F. , Guy, A. , Sébastien, D. T. , Boniface, Y. , Paulin, A. , … Sohounhloue, D. K. C. (2012). Chemical composition and antifungal activity of essential oil of fresh leaves of *Ocimum gratissimum* from Benin against six Mycotoxigenic Fungi isolated from traditional cheese wagashi. Research Journal of Biological Sciences, 1(4), 22–27.

[fsn3888-bib-0027] Philippe, S. , Souaïbou, F. , Paulin, A. , Issaka, Y. , & Dominique, S. (2012). In vitro antifungal activities of essential oils extracted from fresh leaves of *Cinnamomum zeylanicum* and *Ocimum gratissimum* against foodborne pathogens for their use as traditional cheese wagashi conservatives. Research Journal of Recent Sciences, 1(9), 67–73.

[fsn3888-bib-0028] Ramos, Ó. L. , Fernandes, J. C. , Silva, S. I. , Pintado, M. E. , & Malcata, F. X. (2012). Edible films and coatings from whey proteins: A review on formulation, and on mechanical and bioactive properties. Critical Reviews in Food Science and Nutrition, 52(6), 533–552. 10.1080/10408398.2010.500528 22452733

[fsn3888-bib-0029] Renye, J. A. , & Somkuti, G. A. (2015). Bacteriocins of food grade lactic acid bacteria in hurdle technology for milk and dairy products. Emerging Dairy Processing Technologies Opportunities for the Dairy Industry, 267–306. 10.1002/9781118560471

[fsn3888-bib-0030] Ribeiro, D. S. , Siqueira, F. G. , da Silva Velozo, E. , & Guimarães, A. G. (2013). Evaluation rosemary essential oil in the control of multidrug‐resistant *Escherichia coli* in Coalho cheese. Journal of Biotechnology and Biodiversity, 4(1), 1–9.

[fsn3888-bib-0031] Sadeghi, E. , Akhondzadeh Basti, A. , Noori, N. , Khanjari, A. , & Partovi, R. (2013). Effect of *Cuminum cyminum* L. essential oil and *Lactobacillus acidophilus* (a probiotic) on *Staphylococcus aureus* during the manufacture, ripening and storage of white brined cheese. Journal of Food Processing and Preservation, 37(5), 449–455. 10.1111/j.1745-4549.2011.00664.x

[fsn3888-bib-0032] Sadeghi, E. , Mohammadi, A. , Jamilpanah, M. , Bashiri, M. , & Bohlouli, S. (2016). Antimicrobial effects of *Mentha pulegium* essential oil on *Listeria monocytogenes* in Iranian white cheese. Journal of Food Quality and Hazards Control, 3(1), 20–24.

[fsn3888-bib-0033] Seow, Y. X. , Yeo, C. R. , Chung, H. L. , & Yuk, H. G. (2014). Plant essential oils as active antimicrobial agents. Critical Reviews in Food Science and Nutrition, 54(5), 625–644. 10.1080/10408398.2011.599504 24261536

[fsn3888-bib-0034] Sharifi, F. , Khanzadi, S. , Hashemi, M. , & Azizzadeh, M. (2017). Control of *Listeria monocytogenes* and *Escherichia coli* O157:H7 inoculated on fish fillets using alginate coating containing lactoperoxidase system and *Zataria multiflora* boiss essential oil. Journal of Aquatic Food Product Technology, 26(9), 1014–1021. 10.1080/10498850.2017.1375057

[fsn3888-bib-0035] Shin, Y. J. , Song, H. Y. , Seo, Y. B. , & Song, K. B. (2012). Preparation of red algae film containing grapefruit seed extract and application for the packaging of cheese and bacon. Food Science and Biotechnology, 21(1), 225–231. 10.1007/s10068-012-0029-x

[fsn3888-bib-0036] Shokri, S. , Ehsani, A. , & Jasour, M. S. (2015). Efficacy of lactoperoxidase system‐whey protein coating on shelf‐life extension of rainbow trout fillets during cold storage (4°C). Food and Bioprocess Technology, 8(1), 54–62. 10.1007/s11947-014-1378-7

[fsn3888-bib-0037] Smith‐Palmer, A. , Stewart, J. , & Fyfe, L. (2001). The potential application of plant essential oils as natural food preservatives in soft cheese. Food Microbiology, 18(4), 463–470. 10.1006/fmic.2001.0415

[fsn3888-bib-0038] Soleimani‐Rambod, A. , Zomorodi, S. , Naghizadeh Raeisi, S. , Khosrowshahi Asl, A. , & Shahidi, S. A. (2018). The effect of Xanthan gum and flaxseed mucilage as edible coatings in cheddar cheese during ripening. Coatings, 8(2), 80 10.3390/coatings8020080

[fsn3888-bib-0039] Sulieman, A. M. E. , Ohag, O. M. , Hassan, H. M. , Alreshidi, M. M. , & Veettil, E. A. V. N. (2018). Some chemical and microbiological characteristics of Gouda cheese. Advances in Bioresearch, 9(3), 1–6.

[fsn3888-bib-0040] Trmčić, A. , Chauhan, K. , Kent, D. J. , Ralyea, R. D. , Martin, N. H. , Boor, K. J. , & Wiedmann, M. (2016). Coliform detection in cheese is associated with specific cheese characteristics, but no association was found with pathogen detection. Journal of Dairy Science, 99(8), 6105–6120. 27289158 10.3168/jds.2016-11112

[fsn3888-bib-0041] Wemmenhove, E. , Wells‐Bennik, M. H. J. , Stara, A. , Van Hooijdonk, A. C. M. , & Zwietering, M. H. (2016). How NaCl and water content determine water activity during ripening of Gouda cheese, and the predicted effect on inhibition of *Listeria monocytogenes* . Journal of Dairy Science, 99(7), 5192–5201. 10.3168/jds.2015-10523 27085417

[fsn3888-bib-0042] Yener, F. Y. , Korel, F. , & Yemenicioğlu, A. (2009). Antimicrobial activity of lactoperoxidase system incorporated into cross‐linked alginate films. Journal of Food Science, 74(2), M73–M79. 10.1111/j.1750-3841.2009.01057.x 19323761

